# Oral cancer preventive behaviors in rural women: application of the theory planned behavior

**DOI:** 10.3389/froh.2024.1408186

**Published:** 2024-09-05

**Authors:** Fatemeh Mohammadkhah, Amirhossein Kamyab, Ali Khani Jeihooni

**Affiliations:** ^1^ Department of Community Health, Child Nursing and Aging, Ramsar School of Nursing, Babol University of Medical Sciences, Babol, Iran; ^2^ Faculty of Medicine, Fasa University of Medical Sciences, Fasa, Iran; ^3^ Department of Public Health, School of Health, Nutrition Research Center, Shiraz University of Medical Sciences, Shiraz, Iran

**Keywords:** cancer, mouth, behavior, women, cancer prevention

## Abstract

**Background:**

Oral cancer is becoming a primary concern for non-communicable illnesses and global health care initiatives. Low-income people, people with disabilities, the elderly, residents of detached and rural regions, and people belonging to minority groups bear a greater burden of oral diseases. The purpose of this research is to identify rural women's oral cancer prevention activities using the theory of planned behavior (TPB).

**Methods:**

The current research is a cross-sectional analysis of 700 female hookah users who were referred to rural health facilities in Fasa and Shiraz, Fars, Iran in 2019–2020. The participants were selected by random sampling method. The TPB questionnaire and a demographic information questionnaires were the data gathering instruments used in this study to assess oral cancer prevention practices among participants. Data were analyzed by SPSS 22 using frequency, mean, and standard deviation as descriptive statistics, and Pearson correlation coefficients and linear regression as inferential statistics at a significance level of *P* < 0.05. The Kolmogorov-Smirnov test was used to determine whether the data were normal.

**Results:**

The average age of the participants was 44.54 ± 8.72 years, and the average age at which they started hookah was 23.8 ± 28.68 years. The average history of hookah use was 15.8 ± 6.65 years, and the average size of the household in the test group was 4.73 ± 1.16. The average scores of the constructs of the TPB and oral cancer prevention behaviors were average or at a low level, while nicotine addiction was relatively high. The constructs of knowledge, attitude, subjective norms, and behavioral intention are significantly correlated with oral cancer prevention behaviors in hookah-user women. There was a significant inverse relationship between nicotine dependence and oral cancer prevention behaviors, and there was a strong link between perceived behavioral control and behavioral intention (*P* < 0.05).

**Conclusion:**

Based on the results of this study, the average scores of the structures of the TPB and oral cancer prevention behaviors were average or at a low level among rural hookah-user women, which indicates the necessity of an educational program based on this theory for rural women to adopt and maintain oral cancer prevention behaviors.

## Background

Oral cancer, according to the American Joint Committee on Cancer (AJCC) parameters, is a malignant neoplasm originating from the epithelial lining of the oral cavity, classified by tumor size, lymph node involvement, and distant metastasis (1). Based on the Global Cancer Observatory (GLOBOCAN) 2022 data, oral cancer is a significant global health concern with over 389,000 new cases and 188,000 deaths annually, underscoring the need for effective prevention and control strategies (2). Oral cancer is associated with a range of risk factors identified by the World Health Organization (WHO), including tobacco and alcohol consumption, poor nutrition, and certain viral infections such as human papillomavirus (HPV). Other potential risk factors include chronic inflammation, persistent oral irritation, and excessive sun exposure (3). While some of these factors are hereditary and unavoidable, a great majority of them could be improved with taking prevention practices, including healthy eating, avoiding tobacco and alcohol consumption, and safe sexual behaviors.

Hookah smoking, a particularly concerning risk factor, has been linked to an increased risk of oral cancer in several studies (4, 5). The carcinogenic compounds present in hookah smoke, such as polycyclic aromatic hydrocarbons and nitrosamines, contribute to the development of oral cancer through mechanisms including oxidative stress, DNA damage, and inflammation (6–8). Disease or injury that affects oral health might also have an impact on overall health. Pain and suffering brought on by oral diseases make it challenging to concentrate, make people miss their work or school, and can result in social isolation (9). Having a negative impact on people's quality of life, oral and dental disorders have serious social and economic burden on individuals and families (10).

On the other hand, people's knowledge of oral mucosa health and oral cancer is still insufficient and there is still a great need for improvement (11, 12). Sadly, oral and dental diseases mostly affect disadvantaged and underprivileged groups of people (13). People from low socioeconomic backgrounds bear a higher burden of oral and dental problems, and this association endures throughout life, from early childhood to senior age, independent of the nation's total income level (10). Low-income people, those with disabilities, seniors living alone or in nursing homes, residents of isolated and rural areas, and members of minority groups bear a heavier burden of oral diseases (9). Currently, oral cancer is the main priority of non-communicable diseases and international health care plans (14). The WHO supports the implementation of effective population-based preventive interventions and patient-centered care as part of comprehensive programs (15).

However, despite the importance of preventive interventions, there are no programs for prevention of oral cancer in Iran. The incidence of oral cavity cancer in Iran is lower than the global average (1.96 and 1.46 for men and women, respectively); however, considering population aging, increased life expectancy, and exposure to risk factors like smoking, oral cancer incidence is expected to increase in the future (14). According to the statistics, there are roughly 1,400 new cases of oral cancer per 100,000 people per year in Iran (16). In Iran, due to the dominant religious backgrounds, the main risk factor of oral cancer could be attributed to smoking rather than alcohol consumption, especially in southern provinces (17). Hookah use is the most socially-accepted form of smoking especially in rural areas of Iran (18). The most common types of hookahs used in Iran contain fruit-flavored tobaccos, containing high amounts of chemicals, and traditional tobaccos (19). Hookah use has become more popular in women lately (20). According to recent investigations, in southern parts of Iran hookah use has been more prevalent among women rather than men (21) and about 13.98 percent of Iranian women reported using hookahs on average (22).

Given the concerning trends in smoking, evaluating the knowledge, attitude, and oral cancer prevention practices among deprived populations seems necessary, and the information obtained can help in implementing public policies with the aim of continuous oral cancer education. Health education initiatives are required to promote people's knowledge of malignant oral lesions, improve early detection, and recognize the dentist as a leading authority in disease diagnosis (23). The most effective educational programs are based on theory-oriented approaches that are rooted in the models and theories of health education and behavior change psychology. One of the common theories whose efficiency have been confirmed in evaluating various health is called the Theory of Planned Behavior (TPB), which is fundamentally a social cognitive theory of decision-making, which is seen as an efficient theoretical foundation for directing behavior (24).

According to this theory, attitude, subjective norms, and perceived behavioral control are three independent variables that have an impact on intention. A person's attitude reveals whether they think their behavior is positive or negative. The term “subjective norms” describes the perceived social factors that might or might not influence someone to act in a certain way. Perceived behavioral control, which influences behavior both directly and indirectly, is the perceived difficulty or ease of performing a specific behavior. According to the concept of TPB, people will intend to engage in a behavior if they find it positive, come to believe that others find it necessary, and assume that they have control over it (25). This theory has been used to predict and describe behaviors related to smoking, drinking, and drug abuse, and the model structures predict a significant percentage of the variance of intention and behavior (26).

Identifying factors affecting the adoption and continuation of oral cancer preventive behaviors, which is mostly focused on alcohol and tobacco consumption and safe sexual behavior, using theories and behavior change models is accepted as a necessity before designing preventive cognitive and behavioral interventions. Considering the prevalence of oral cancer in Iran, the lack of knowledge and access of women living in remote and underprivileged areas to health services, the role of the TPB in predicting behavior, and the prevalence of hookah smoking among Iranian women, the present study was conducted with the aim of determining oral cancer prevention behaviors in 700 deprived hookah smoker women using the TPB.

## Methods

### Research design

This cross-sectional study was conducted on 700 female hookah smokers who were referred to rural health centers in Fasa and Shiraz, Fars, Iran, in 2019–2020. Out of the total 20 rural health centers in Fasa and Shiraz, two centers were chosen using a simple random sampling method. Each center was assigned a number, and a random number generator was used to select the centers. Then, based on the inclusion criteria of the study, 700 female hookah smokers were contacted and invited to participate in the study and attend the health center. In accordance with a former study by Bommireddy et al. (27), the sample size was calculated using the formula “*n* = (1.96)^2^*P*(1−*p*)/*d*^2^” as 700 people.

### Inclusion and exclusion criteria

The study included females who were over 15 years old, used hookah at least once a day for a minimum of one year, and provided written agreement to participate. Participants with psychological or physical illnesses were excluded from the study. The determination of psychological illness was made based on self-reported information from the participants and verified by a brief screening questionnaire conducted by a trained healthcare professional. Additionally, participants who did not fully complete the questionnaire were excluded from the study.

### Data collection process

Participants were contacted by trained healthcare professionals through the rural health facilities they visited. A gatekeeper, typically a nurse or health center staff member, facilitated initial contact and introduced the study to potential participants, ensuring that those who met the inclusion criteria were informed and invited to participate. In this study, all individuals provided informed consent. Participants under the age of 18 provided explicit parental or guardian consent before being included in the study.

In this study, data collection tools were paper-based questionnaires including a demographic information questionnaire and the TPB questionnaire. The demographic information questionnaire included age, the age of starting hookah use, the average duration of hookah use, monthly household income, educational level, marital status, history of oral cancer in the family, history of cigarette smoking, history of alcohol consumption, history of hookah use in the family, and the type of hookah used. The second part of questions which was related to the TPB, included questions about knowledge, attitude, subjective norms, perceived behavioral control, behavioral intention, and behavior. Questionnaires were completed either individually, or with the assistance of a researcher (for illiterate participants), and the validity and reliability of questionnaires have been confirmed in other studies (28–33).

The TPB consisted of 15 questions pertaining to knowledge on hookah smoking, associated complications, and resulting diseases. A score of 1 was assigned to the correct answer, while an incorrect answer or “I don't know” received a score of 0. The total score ranged from 0 to 15.

The attitude was assessed using a 5-point Likert scale consisting of 12 questions, ranging from “completely disagree” to “completely agree”. The score was determined using a range of values from 12 to 60. The construct of perceived behavioral control was assessed using a Likert scale consisting of 10 questions. The scale ranged from “completely disagree” to “completely agree” with a scoring range of 10 to 50. The subjective norms construct comprised of eight questions that assessed the individual's perception of support from various sources such as family, friends, other dependents, and health workers. The responses were measured using a Likert scale consisting of 5 levels ranging from 0, indicating “not at all,” to 4, indicating “very much.” The total score ranged from 0 to 32. The behavioral intention was assessed using a set of 12 dichotomous questions, with response options of either “yes” or “no”. The scores ranged from 0 to 12. The survey on oral cancer prevention habits consisted of 12 questions with yes or no alternatives. The scores ranged from 0 to 12.

Lastly, the Schiffman Nicotine Dependence Scale (NDSS) was used to quantify nicotine dependence through the use of 19 questions. All the questions on this scale were scored based on a 5-point Likert scale “Not at all true” to “Completely true”; the minimum score was 19 and the maximum score was 95, where higher scores indicate greater dependence on nicotine (34). It took two months for the researchers to distribute and collect the questionnaires. Finally, data was entered to SPSS version 22 software for further analysis.

### Validation process and handling of missing data

The TPB questionnaire utilized in this study underwent a rigorous validation process to ensure its reliability and validity. Initially, the questionnaire was developed based on established TPB constructs and reviewed by a panel of experts in behavioral science and public health to ensure content validity. A pilot study was then conducted with a small sample of participants (*n* = 50) from the target population to test the clarity, relevance, and comprehensiveness of the questions. Feedback from the pilot study was used to refine the questionnaire. Construct validity was assessed using exploratory factor analysis (EFA) to confirm the underlying theoretical structure of the TPB constructs. Additionally, internal consistency was evaluated using Cronbach's alpha coefficients, with values exceeding the acceptable threshold of 0.70 for all constructs, indicating high reliability.

Regarding the handling of missing data, we implemented several strategies to minimize its impact on the study's findings. During data collection, efforts were made to ensure complete responses by training data collectors to assist participants in understanding and answering all questions. For any remaining missing data, we employed multiple imputation methods to handle missing values appropriately. This technique allowed us to generate plausible values based on the observed data, thus maintaining the integrity and statistical power of our analyses. Cases with excessive missing data (more than 20% of the questionnaire) were excluded from the analysis to prevent bias. These steps ensured the robustness and reliability of our study results.

### Ethical considerations

The Shiraz University of Medical Sciences ethics committee gave its approval for this study under the ethics committee code “IR.SUMS.SCHEANUT.REC.1401.110”. Participants provided their informed consent by completing a consent form. For participants under the age of 18, parental or guardian consent was explicitly obtained, ensuring compliance with ethical standards for involving minors in research. The participants were provided with a clear explanation of the study's objectives, importance, and necessity. Additionally, they were assured that their information would be kept confidential.

### Data analysis

The normality of the data was measured by the Kolmogorov-Smirnov test. To describe oral cancer prevention behaviors in participants, descriptive statistics (frequency, mean, and standard deviation) were used. The relationship between the structures of TPB and nicotine dependence was determined by Pearson correlation coefficients. And linear regression was applied to predict oral cancer prevention behaviors among female hookah users. The significance level was considered to be *P* < 0.05.

## Results

In this study, 700 women were examined. The average age of the examined women was 44.54 ± 8.72 years, the average age of starting hookah use was 23.8 ± 28.68 years, the average history of hookah use was 15.8 ± 6.65 years, and the average size of the household in the test group was 4.73 ± 1.16. Other demographic characteristics of the subjects are given in Table 1.

**Table 1:** The distribution of the subjects’ demographic characteristics.

Variable		Frequency	Percentage
Household income	Low	366	52.29%
	Average	268	38.28%
	High	66	9.43%
Educational level	Illiterate	28	4%
	Elementary school	171	24.43%
	Secondary school	234	33.43%
	High School	232	33.14%
	University degree	35	5%
Marital status	Single	78	11.14%
	Married	573	81.86%
	Divorced	27	3.86%
	Widowed	22	3.14%
Family history of oral cancer	Yes	26	3.71%
	No	674	96.29%
History of smoking	Yes	442	63.14%
	No	258	36.86%
History of alcohol consumption	Yes	63	9%
	No	637	91%
History of hookah use in the family	Yes	641	91.57%
	No	59	8.43%
Type of hookah used	Fruit hookah	316	45.14%
	Traditional hookah	384	54.86%

Table 2 shows the average score of the constructs of the TPB and oral cancer prevention behaviors, which are often average or at a low level, while nicotine addiction is at a high level.

**Table 2:** Comparison of the TPB, oral cancer prevention behaviors, and the level of nicotine dependence in the studied subjects.

Variable	Mean	SD	Minimum	Maximum
Knowledge	7.67	0.56	0	15
Attitude	26.33	3.78	12	60
Perceived behavioral control	24.28	3.59	10	50
Subjective norms	10.12	2.41	0	32
Behavioral intention	4.80	0.78	0	12
Oral cancer prevention behaviors	4.25	0.52	0	12
Nicotine dependence level	80.44	5.90	19	95

The results of Table 3 show that knowledge, attitude, subjective norms, and behavioral intention have a significant direct relationship with oral cancer prevention behaviors in rural women who use hookah. There is a significant inverse relationship between nicotine dependence and oral cancer prevention behaviors. There is no significant relationship between perceived behavioral control and behavioral intention, but there is no significant relationship with oral cancer prevention behaviors.

**Table 3:** Correlation between the variables studied on the oral cancer prevention behaviors in women.

Variables	Knowledge	Attitude	Perceived behavioral control	Subjective norms	Behavioral intention	Nicotine dependence level	Oral cancer prevention behaviors
Knowledge	1	0.17*	0.15*	0.08	0.31*	0.21*	0.19*
Attitude	0.80*	1	0.42*	0.14*	0.28*	0.16*	0.24*
Perceived behavioral control	0.24*	0.31*	1	0.26	0.23*	0.25	0.20
Subjective norms	0.34	0.22*	0.24	1	0.28*	0.17*	0.28*
Behavioral intention	0.25*	0.21*	0.23*	0.45*	1	0.29*	0.22*
Nicotine dependence level	0.34*	0.20*	0.26	−0.38*	−0.30*	1	−0.33*
Oral cancer prevention behaviors	0.21*	0.23*	0.29	0.27 *	0.19*	0.27*	1

* *p* < 0.05.

According to Table 4, linear regression showed that knowledge, attitude, subjective norms, nicotine dependence, and behavioral intention predicted oral cancer prevention behaviors among female hookah users. In general, the variables predicted 63.6% of oral cancer prevention behaviors.

**Table 4:** Analysis of factors related to oral cancer behaviors.

Variables	Beta	S.E	B	*p*	Change
Knowledge	0.218	0.74	0.108	0.024	Oral cancer prevention behaviors $R^{2}=0.636$ $R^{2}$*Adjusted = 0.182*
Attitude	0.221	0.79	0.124	0.020	
Perceived behavioral control	0.181	0.67	0.122	0.118	
Subjective norms	0.205	0.84	0.128	0.028	
Nicotine dependence level	−0.212	0.90	−0.131	0.031	
Behavioral intention	0.244	0.81	0.136	0.018	

## Discussion

The present study was conducted with the aim of determining the oral cancer prevention behaviors of rural women. According to the results of the present study, rural women's knowledge of oral cancer prevention behaviors was at an average level. This finding is consistent with the results of studies by Razavi et al., Shubayr et al., Saraswat et al., and Petrauskienė et al. (35–38). This finding could be due to the lack of an organized educational program for raising knowledge of this population regarding oral cancer and its prevention. This information emphasizes the need to develop comprehensive written education programs in this field. Obviously, by spreading educational content, rural women's knowledge regarding oral cancer will rise considerably (39).

Based on our results, rural women's attitudes towards oral cancer prevention behaviors were at a low level, which is in line with the results of studies by Shubayr et al., Shwetha et al., and Baumann et al. (36, 40, 41). In this study, low attitude levels meant that rural women did not have a bright perception of smoking. A person's attitude is defined as their knowledge, emotions, and willingness to take action toward a particular object. Attitude is a relatively stable desire for a person, something, or an event that manifests itself in feelings and behavior (42, 43). By knowing the positive and negative attitudes, examining the factors influencing their formation, and formulating family-oriented and targeted educational programs, attitudes can be guided, which can lead to changes in behaviors. Therefore, educational interventions formed on strengthening the attitudes of oral cancer prevention behaviors, can take an effective step to promote the adoption of these behaviors.

According to the results, perceived behavioral control in rural women regarding oral cancer prevention practices was at an average level, which is consistent with the results of studies by Shwetha et al., Hossein et al., and Alami et al. (40, 44, 45). Perceived behavioral control reflects a person's perception of the ease or complexity of an action as well as their level of control over it (43). Whenever people feel they have enough resources, self-confidence, and ability to perform a certain behavior, they will take an action (46). Probably, our studied population was less likely to have a strong intention to engage in oral cancer prevention behaviors since they believed they lacked the required resources or self-esteem. By carrying out interventions in the form of educational sessions aimed at improving rural women's self-confidence and empowerment, improving access to resources, reducing barriers, and improving the subsequent perceived behavioral control, an effective step can be taken in adopting and continuing oral cancer prevention practices in this population. It is noteworthy that different prevention approaches are needed for different groups with different educational levels, and more focus should be on comprehensive educational programs.

In this study, the subjective norms of rural women regarding oral cancer prevention behaviors was at a low level. These finding were consistent with the results of studies by Moghaddam et al., Mohammadi Zeidi et al., and Alami et al. (26, 44, 47). Subjective norms are perceived pressure from others to engage in certain activities or not, and reflect the individual's desire to follow others (43). People are influenced by various people in society, and base their intentions on the wishes of others (48). People are influenced by the behavior, personality, and thoughts of those because them gives them a sense of validity and independence. In this population, due to the lack of a proper education and because of the existence of false norms in the society, the ground for deviations towards other risky behaviors are provided. Conclusively, educating and encouraging friends and family members to support the individuals plays an important role in their prevention practices. In this case, training sessions along with getting support from family and friends could be very helpful in improving oral cancer prevention behaviors.

The results of this study revealed that behavioral intention of rural women regarding oral cancer prevention behaviors was low, which is in good agreement with the results of studies by Nigatu et al., Alami et al., and Mohammadi Zeydi et al. (26, 48, 49). Probably, the reason behind this finding was low levels of attitudes, subjective norms, and perceived behavioral control, which have a direct relationship with behavioral intention. The most crucial aspect in defining a person's behavior is behavioral intention, and a person may engage in a certain action as a result of a mix of attitudes, subjective norms, and perceived behavioral control (44). Considering the role of behavioral intention in performing preventive behaviors, it is recommended to prepare and implement training programs for rural women to promote behavioral intention.

Our results also showed that the scores of oral cancer prevention behaviors in rural women were dissatisfying, which is in line with the results of studies of Shubayr et al.'s, Shadid et al., and Ahmed et al. (36, 50, 51). As mentioned earlier, prevention behaviors are the results of guided attitudes, subjective norms, perceived behavioral control, and behavioral intention. Obviously with the spread of inaccurate attitudes, wrong subjective norms, and false perceived behavioral control, preventive health behaviors cannot be expected. These findings emphasizes on the need for conducting well-planned educational program in this regard.

In accordance with the results, the constructs of the TPB had a significant relationship with each other, which is consistent with the results of the studies of Hamilton et al. and Pundir et al. (52, 53). Considering the relationship between the structures of this model and the fact that in this study, the levels of all the structures of this model were at a low and medium level, it can be expected that the level of oral cancer prevention behaviors in rural women participating in the present study is also low. Therefore, carrying out an effective educational intervention to improve oral cancer prevention behaviors seems necessary.

Ultimately, the rate of nicotine dependence among the participants was relatively high. Nicotine dependence among the participants in the studies of Alami et al. and Saeed Firoozabadi et al. were at an alarming rate (48, 54). It is necessary to design educational interventions for people of low socio-economic class, especially in adolescents when the behavior is not yet institutionalized. Moreover, for people who don't intend to quit hookah, the conditions for them to quit must be prepared through the empowerment processes, that encourages them to quit hookah.

### Strengths and weaknesses

One of the limitations of the present study was the use of self-reported questionnaire tools in data collection, which can introduce self-report biases such as social desirability bias and recall bias. Participants may have over-reported socially acceptable behaviors or under-reported undesirable behaviors like hookah use. Additionally, the reliance on self-reported data for psychological illnesses may not have captured all relevant conditions accurately. Future studies should consider incorporating objective measures, such as biochemical verification of smoking status, or validated diagnostic tools for psychological illnesses, to minimize these biases. Another limitation was the potential for selection bias, as the study only included women who were willing to visit the health centers and participate in the research. This might exclude more isolated or less health-conscious individuals, potentially skewing the results.

Among the strengths of the present study, we can mention the investigation of oral cancer prevention behaviors based on the TPB theory, which provides a robust framework for understanding health behaviors. The study also targeted a specific high-risk group (rural women) that is often underserved in health interventions, emphasizing the importance of tailored public health strategies. Additionally, the random selection of health centers and the large sample size (700 participants) contribute to the generalizability of the findings. However, it is essential to address the identified limitations to enhance the validity and reliability of future research in this area.

## Conclusion

Based on the results of the present study, the average scores of knowledge, attitude, perceived behavioral control, subjective norms, behavioral intention, and oral cancer prevention behaviors among rural female hookah users were at a low to average level. Additionally, nicotine addiction was at a high level among this population. The constructs of knowledge, attitude, subjective norms, and behavioral intention had a significant direct relationship with oral cancer prevention behaviors. Interestingly, there was a significant inverse relationship between nicotine dependence and oral cancer prevention behaviors. Although lifestyle changes are successfully achieved with professional assistance and pharmacotherapy, implementing educational interventions in order to boost knowledge, attitude, and prevention of oral cancer could be effective in the future, especially in underprivileged populations.

In accordance with the findings of the present study, efficient educational programs addressing the components of the TPB could be implemented using educational contents on television networks and social media platforms to raise public knowledge about the etiology of oral cancer and the harms of smoking, and take effective steps in oral cancer prevention behaviors.

## Data Availability

The raw data supporting the conclusions of this article will be made available by the authors, without undue reservation.
