# Supplementary material for: Oral cancer preventive behaviors in rural women: application of the theory planned behavior
Source: Front Oral Health. 2024 Sep 5;5:1408186. doi: 10.3389/froh.2024.1408186 (PMC11410771; doi:10.3389/froh.2024.1408186)
Supplement: Supplementary file 1 [file Datasheet1.pdf]

Demographic Information Questionnaire

1. Age: _______
2. Household income:
   - Low
   - Average
   - High
3. Educational attainment:
   - Illiterate
   - Elementary school
   - Secondary school
   - High School
   - University degree
4. Marital status:
   - Single
   - Married
   - Divorced
   - Widowed
5. Family history of oral cancer:
   - Yes
   - No
6. Smoking history:
   - Yes
   - No
7. Alcohol consumption history:
   - Yes
   - No
8. Family history of hookah use:

- Yes
- No

9. Type of hookah used:
   - Fruit hookah
   - Traditional hookah

Theory of Planned Behavior Questionnaire

Knowledge on Hookah Smoking Complications and Resulting Diseases

(Score 1 for correct answer, 0 for incorrect or "I don't know")

1. Hookah smoking can cause oral cancer. (True/False/I don't know)
2. Hookah use is less harmful than cigarette smoking. (True/False/I don't know)
3. Hookah smoking can lead to respiratory diseases. (True/False/I don't know)
4. Hookah use does not affect cardiovascular health. (True/False/I don't know)
5. Using hookah in social gatherings is safe. (True/False/I don't know)
6. Hookah smoke contains toxic substances. (True/False/I don't know)
7. Sharing hookah mouthpieces can spread infectious diseases. (True/False/I don't know)
8. Hookah smoking can cause addiction. (True/False/I don't know)
9. Pregnant women should avoid using hookah. (True/False/I don't know)
10. Hookah use can affect oral hygiene. (True/False/I don't know)
11. Hookah smoking can lead to gum disease. (True/False/I don't know)
12. The water in hookah filters out harmful chemicals. (True/False/I don't know)
13. Hookah use can impair lung function. (True/False/I don't know)
14. Hookah smoking can lead to throat cancer. (True/False/I don't know)
15. Hookah smoke is less irritating than cigarette smoke. (True/False/I don't know)

Attitude (5-point Likert scale: 1 = Completely disagree, 5 = Completely agree)

1. Hookah smoking is harmful to my health.

2. I believe quitting hookah will improve my quality of life.

3. Hookah smoking is a relaxing activity.

4. I enjoy smoking hookah with friends.

5. Hookah smoking is not as dangerous as other forms of smoking.

6. I worry about the long-term effects of hookah smoking.

7. Hookah smoking makes me feel more social.

8. Quitting hookah would be beneficial for my health.

9. Hookah use is an acceptable social activity.

10. I feel guilty about smoking hookah.

11. Hookah smoking is a good way to relieve stress.

12. I am concerned about the health risks of hookah smoking.

Perceived Behavioral Control (5-point Likert scale: 1 = Completely disagree, 5 = Completely agree)

1. I have control over my hookah smoking habits.

2. It is easy for me to avoid using hookah when I want to.

3. I can quit hookah smoking if I want to.

4. I am confident in my ability to quit hookah smoking.

5. I have the resources to quit hookah smoking.

6. I have the support needed to quit hookah smoking.

7. Quitting hookah smoking is within my control.

8. I find it difficult to quit hookah smoking.

9. I can resist the temptation to smoke hookah.

10. I am capable of quitting hookah smoking.

Subjective Norms (5-point Likert scale: 0 = Not at all, 4 = Very much)

1. My family supports my decision to quit hookah.

2. My friends think I should quit using hookah.

3. My healthcare provider encourages me to quit hookah.

4. My community supports efforts to quit hookah.

5. My spouse/partner supports my decision to quit hookah.

6. My children want me to quit hookah.

7. My religious or spiritual beliefs discourage hookah use.

8. Health campaigns in my area encourage quitting hookah.

Behavioral Intention (Yes/No)

1. Do you intend to quit using hookah within the next month?

2. Do you plan to seek help to quit hookah use?

3. Will you try to reduce the number of times you use hookah each week?

4. Do you intend to quit using hookah within the next six months?

5. Are you planning to participate in a hookah cessation program?

6. Will you avoid social gatherings where hookah is used?

7. Do you intend to talk to a healthcare provider about quitting hookah?

8. Are you planning to replace hookah with healthier activities?

9. Do you intend to inform your friends about the dangers of hookah smoking?

10. Will you set a quit date for hookah smoking?

11. Do you plan to use nicotine replacement therapy to quit hookah?

12. Are you planning to avoid places where hookah is sold?

Oral Cancer Prevention Behaviors (Yes/No)

1. Do you regularly check your mouth for any unusual sores or lumps?

2. Have you ever sought medical advice about the risks of hookah smoking?

3. Do you use any protective measures to prevent oral cancer?

4. Have you attended any educational sessions on oral cancer prevention?

5. Do you follow a healthy diet to reduce the risk of oral cancer?

6. Do you avoid using tobacco products other than hookah?

7. Do you limit your alcohol consumption to reduce cancer risk?

8. Have you had regular dental check-ups in the past year?

9. Do you avoid exposure to secondhand smoke?

10. Do you maintain good oral hygiene practices?

11. Do you avoid using shared hookah mouthpieces?

12. Have you encouraged others to avoid hookah smoking?

Nicotine Dependence (Schiffman Nicotine Dependence Scale - NDSS) (5-point Likert scale: 1 = Not at all true, 5 = Completely true)

1. I often crave hookah when I can't use it.

2. I find it difficult to go a day without using hookah.

3. I use hookah more frequently than I initially intended.

4. I feel restless when I cannot use hookah.

5. I use hookah to cope with stress.

6. I spend a lot of time thinking about hookah.

7. I have tried to cut down on hookah use but failed.

8. I feel a strong need to use hookah after a few hours without it.

9. I prefer using hookah over other recreational activities.

10. I find it difficult to concentrate without using hookah.

11. I feel irritable when I can't use hookah.

12. I use hookah to avoid withdrawal symptoms.

13. I have increased my hookah use over time.

14. I feel a sense of relief when I use hookah.

15. I have continued using hookah despite knowing its harms.

16. I plan my day around opportunities to use hookah.

17. I have used hookah in situations where it is not allowed.

18. I often use hookah as soon as I wake up.

19. I have experienced physical symptoms when I haven't used hookah for a while.
